# Patient involvement in treatment decisions is associated with increased therapy satisfaction in Hidradenitis suppurativa

**DOI:** 10.3389/fmed.2025.1626345

**Published:** 2025-09-03

**Authors:** Giorgia Cugno, Sylke Schneider-Burrus, Georgios Kokolakis, Dagmar Wilsmann-Theis, Katharina Assaf, Rotraut Moessner, Christian Kromer, Falk G. Bechara, Nessr Abu Rached, Wiebke K. Peitsch, Lisa C. Schneider, Andreas Happ, Valentina Siddi, Diana Kubitzki, Durdana Groß, Markus Friedrich, Staffan Vandersee, Khusru Asadullah, Robert Sabat, Kerstin Wolk

**Affiliations:** ^1^Charité—Universitätsmedizin Berlin, corporate member of Freie Universität Berlin and Humboldt-Universität zu Berlin, Translational Skin Inflammation Research and former Psoriasis Research and Treatment Center, Department of Dermatology, Venereology and Allergology, Berlin, Germany; ^2^Dermatology Practice Dr. Friedrich/Dr. Philipp, Oranienburg, Germany; ^3^Department of Dermatology and Phlebology, Vivantes Klinikum im Friedrichshain, Berlin, Germany; ^4^Centre for Dermatosurgery, Havelklinik, Berlin, Germany; ^5^Centre of Skin Diseases, University Hospital Bonn, Bonn, Germany; ^6^Department of Dermatology, Venereology, and Allergology, University Medical Center, Göttingen, Germany; ^7^International Centre for Hidradenitis Suppurativa/Acne Inversa (ICH), Department of Dermatology, Venereology and Allergology, Ruhr-University Bochum, Bochum, Germany; ^8^Department of Dermatology, Klinikum Frankfurt (Oder), Frankfurt an der Oder, Germany; ^9^Dermatology Practice Siddi & Bachmann, Berlin, Germany; ^10^Medizinisches Versorgungszentrum/Medical Care Center (MVZ) Lobetal, Bernau bei Berlin, Germany; ^11^Dermatology Practice Dr. Gross, Potsdam, Germany; ^12^Department of Dermatology, Bundeswehr Hospital, Berlin, Germany; ^13^Dermatology Potsdam Medizinisches Versorgungszentrum/Medical Care Center (MVZ), Potsdam, Germany

**Keywords:** patient involvement, patient preference, therapy decision, shared decision-making, satisfaction, psoriasis, Hidradenitis suppurativa, patient-centered care

## Abstract

**Background:**

Hidradenitis suppurativa (HS) is a painful and disfiguring chronic inflammatory skin disease. Despite many efforts over the past decade to improve the care of patients with HS, their satisfaction with medical care remains limited.

**Aim:**

The aim of this study was to assess the perceived involvement of patients with HS and, for comparison, patients with psoriasis, in treatment decision-making and to identify areas associated with positive perception.

**Methods:**

A prospective, cross-sectional, questionnaire-based survey was conducted between May 2023 and July 2024 in different types of dermatological care facilities in Germany. Patients rated their therapy decision involvement and treatment satisfaction on a 0–10 scale. Data were stratified by demographic, clinical, and healthcare-related variables.

**Results:**

124 HS patients and 133 psoriasis patients completed the questionnaires. The percentage of HS patients rating their therapy decision involvement as low (values of 0–5) was 27.2%, while the percentage of psoriasis patients who gave the same assessment was 11.9% (*P* < 0.01). Moreover, the average degree of perceived therapy decision involvement was significantly lower for patients with HS compared to psoriasis patients (mean ± SD: 7.0 ± 2.9 vs. 8.4 ± 2.1; *P* < 0.001). Greater involvement in therapy decisions was linked to higher satisfaction of patients with the therapies received (*P* < 0.01). Younger HS patients (18–40 years) reported lower involvement scores (*P* < 0.01), while gender, education level, disease duration, disease severity, number of comorbidities, type of healthcare facility, and type of therapies undergone had no influence. Extended consultation times with the dermatologist (≥20 min; *P* < 0.05) and more than one quarterly visit to the dermatologist (*P* < 0.01) were marginally associated with greater patient involvement in decision-making, but did not explain the difference between patients with HS and psoriasis in this regard. Waiting time until first visit to a dermatologist (negative association, *P* < 0.01) and, more strongly, satisfaction with information provided by the dermatologist about patient's skin disease (positive association, *P* < 0.001) were associated with patient involvement in therapy decision and were significantly different in patients with HS vs. psoriasis.

**Conclusions:**

This study shows limited involvement of HS patients in the therapy decision-making process, which was associated with low treatment satisfaction. Improvement may be achieved by training dermatologists in disease mechanisms and patient communication.

## 1 Introduction

Involvement of patients in therapy decision (also referred to as “shared decision-making”) is the key element in patient-centered care. The objective is to identify the therapeutic intervention for the patient that is supported by the strongest available medical evidence and that best meets the patient's expectations in terms of effectiveness, potential side effects, and method of application (1). Involvement in therapy decision has been shown to improve patient's adherence to treatment (2). It is particularly relevant for complex, long-lasting diseases with different treatment options, including chronic diseases. While long practiced in fields like oncology and cardiology (2), the concept of shared decision-making is less implemented in routine practice in dermatology (3).

Hidradenitis suppurativa (HS) is a chronic inflammatory skin disease with an enormous unmet medical need, affecting ~1% of the population globally (4). It manifests as recurrent or persistent, painful inflammatory nodules and abscesses in the intertriginous skin of the armpits, groin, buttocks and perianal region (4). In later stages, fistulas can form in these regions and extensive scarring can occur. Based on the progressive nature the disease, three disease phases can be distinguished: the inflammatory phase (characterized by reversible inflammatory lesions, such as nodules and abscesses), the destructive phase (characterized by both reversible inflammatory lesions and irreversible skin changes, including single or interconnected tunnels), and the burnout phase (characterized by irreversible lesions such as hypertrophic scars, fibrotic bands, hardened plaques, and contractures, in the absence of relevant signs of inflammation) (4).

In addition to skin changes, a significant proportion of patients suffer from neuropathic pain (5), depression and anxiety (6), extracutaneous inflammatory conditions such as arthritis and intestinal inflammation (7–9) as well as metabolic syndrome and its consequences (10, 11). The latter plays a major role in the recently reported reduction in life expectancy of HS patients by an average of 15 years (12).

Smoking, obesity and a genetic predisposition are considered to be important etiological factors for HS onset (13–15). However, little is known about the exact pathophysiological mechanisms in HS. The disease starts at the terminal hair follicles in the skin folds and leads to a complex inflammatory cascade involving neutrophilic granulocytes and other myeloid cells, lymphoid cells including Th1, Th17, NK, and B cells, as well as their secreted inflammatory mediators (16–23).

In the last decade, many national and international efforts have been made to increase awareness of HS and the medical care of those affected. However, treating HS remains very challenging because of the phase nature of the disease, with each phase requiring a different treatment. Patients with only reversible inflammatory lesions should be primarily treated with medication. Those in the destructive phase, who have both inflammatory lesions and irreversible skin changes, require a combination of medication and surgery. For patients in the burnout phase, the lesions should be removed or the region should be completely excised (4). The first biologic therapies targeting inflammation have been approved for Europe and the USA and show at least moderate efficacy: the anti-TNF-α drug adalimumab and the anti-IL-17 drugs secukinumab (against IL-17A) and bimekizumab (against IL-17A and IL-17F) (4, 24). However, a number of other drugs that are not specifically approved for HS and are based on little scientific evidence are in widespread use (25). These include antibiotics (e.g., doxycycline) (26, 27), hormone-related drugs (e.g., spironolactone) and steroids for intralesional injection (e.g., triamcinolone) (28). The consequence of the fact that drugs are often applied too late in the course of the disease and/or show insufficient effectiveness is that the disease progresses and surgical intervention becomes necessary (29–32). All of this can contribute to poor psychosocial wellbeing and poor treatment satisfaction, leading patients with HS to feel that their disease is not under control (33, 34). It is not surprising that overall satisfaction with medical care for HS is still limited among both HS patients and dermatologists, even in countries with relatively good medical standards such as Germany (35). In addition to the treatment of the skin lesions themselves, the comorbidities of HS patients should also be considered in medical care; however, this remains a theoretical recommendation for now (4).

Given the importance of shared decision-making and the complexity of HS and its treatment options, it is important to investigate to what extent patients with HS currently feel involved in the therapy decision and to identify approaches for improvement. Because nothing is known about this specific topic, we conducted a multi-center survey in Germany. As a comparison to HS patients, we included patients with psoriasis, a disease with a well-met medical need in Western countries. Key clinical, demographic, and healthcare-related parameters, such as patient age, gender, and education, type of care facility, frequency and duration of dermatological consultations, treatment experience, and sources of information about the disease were included for subgroup and association analyses.

## 2 Materials and methods

### 2.1 Study populations

We performed a cross-sectional, prospective, anonymous, observational, questionnaire-based study involving patients diagnosed with either HS or psoriasis vulgaris (35). Participants were recruited from three types of healthcare settings in Germany: independent dermatological practices, dermatological departments of municipal and military hospitals, and dermatological clinics of university hospitals. Inclusion criteria for patients comprised a confirmed diagnosis of HS or psoriasis vulgaris, their consent to participate in the study, and their ability to complete the questionnaire independently. Exclusion criteria included age under 18 years.

The study was approved by the local ethics committee of the Charité–University Medicine (reference number: EA4/205/22, positive vote from February 8, 2023) and was registered in the German Register of Clinical Trials (identification number: DRKS00031572).

### 2.2 Questionnaire design

Questionnaires were designed and used to collect data: one on HS to be completed by the HS patients and one on psoriasis to be completed by the psoriasis patients. These questionnaires were designed in three steps: (i) creation of the first version based on the tool for patient survey (“ZAP”), available on the website of the National Association of Statutory Health Insurance Physicians (Kassenärztliche Bundesvereinigung; https://www.kbv.de/), adapted to our study objective and expanded to include information from treating dermatologists, according to our expertise (34, 36); (ii) review of the first version by physicians from different care facilities; (iii) generation of the final version based on physicians' feedback.

### 2.3 Data collection

Data were anonymously collected via the disease-specific (HS or psoriasis) questionnaires described above. Patients visiting the dermatological care facilities, fulfilling the inclusion criteria and voluntarily agreeing to participate were given the questionnaires, with the first part filled by the treating dermatologist with information about patient's disease severity and therapy experience over the past 12 months. Using these questionnaires, patients then anonymously self-reported their perceived involvement in therapy decision-making by the dermatologist on a 0–10 scale, where 0 indicated no involvement and 10 indicated full involvement. Additionally, they answered questions about demographic aspects, their disease and disease history, and their experiences with the medical care, including their satisfaction with received therapies (topical, systemic, surgical; on 0–10 scales). Data were extracted into predesigned data tables containing pre-defined follow-up calculations.

### 2.4 Statistical analysis

Statistical calculations were made in SPSS Statistics software (IBM), version 27. Two-tailed Mann–Whitney *U*-test, Pearson χ^2^ test, and Spearman correlation test were applied as indicated. *P*-values < 0.05 were considered to indicate significance. Missing data were not filled in. Graphs were prepared using GraphPad Prism software (GraphPad Software, LLC), version 8.4.3.

## 3 Results

### 3.1 HS patients feel poorly involved in the decision regarding the therapy of their skin disease compared to psoriasis patients

Forty German healthcare facilities, including independent dermatological practices, dermatological departments of municipal and military hospitals, and dermatological clinics of university hospitals, were initially contacted for study participation. Questionnaires were sent to the 20 of them who agreed to participate.

A total of 125 HS questionnaires and 133 psoriasis questionnaires were received from the 20 centers. Ultimately, 124 HS questionnaires and 133 psoriasis questionnaires were included in the analysis (one HS questionnaire was not taken into account as it contained answers about psoriasis). The characteristics of the patients included in the analysis are presented in Table 1 (based on the information provided by their dermatologists and by the patients themselves, see the Methods Section).

**Table 1:** Demographic and clinical characteristics of patients participating in this study.

**Patients' features**	**HS**	**Psoriasis**
**Number of patients**		
Total	124	133
From dermatological practices	28	40
From dermatological departments in municipal hospitals	26	26
From University dermatology clinics	59	66
No information provided about the dermatological care facility	1	1
**Patients' age (estimation** ^*^ **)**		
Mean ± SD (range), in years	43.0 ± 12.5 (19–65)	50.7 ± 15.1 (19–75)
18–40 years old (%)	43.1	27.1
41–60 years old (%)	48.0	39.8
61–80 years old (%)	8.9	33.1
**Patients' gender**		
Female (%)	56.9	39.1
Male (%)	43.1	60.9
Diverse (%)	0.0	0.0
**Patients' BMI**		
Mean ± SD (range), in kg/m^2^	31.1 ± 6.2 (18.7–51.6)	29.2 ± 5.6 (14.7–44.8)
**Patients' disease severity**		
IHS4, mean ± SD (range)	17.4 ± 19.8 (0.0–95.0)	
PASI, mean ± SD (range)		6.5 ± 8.9 (0.0–39.8)
**Number of patients treated with biologics in the last 12 months**		
In %	31.4	55.7

^*^Calculated on the basis of the surveyed mean value of each of the age ranges (18–20, and third to seventh decade of age).

HS, Hidradenitis suppurativa; BMI, body mass index; IHS4, international Hidradenitis suppurativa severity scoring system; PASI, psoriasis area and severity index.

**Figure 1:**
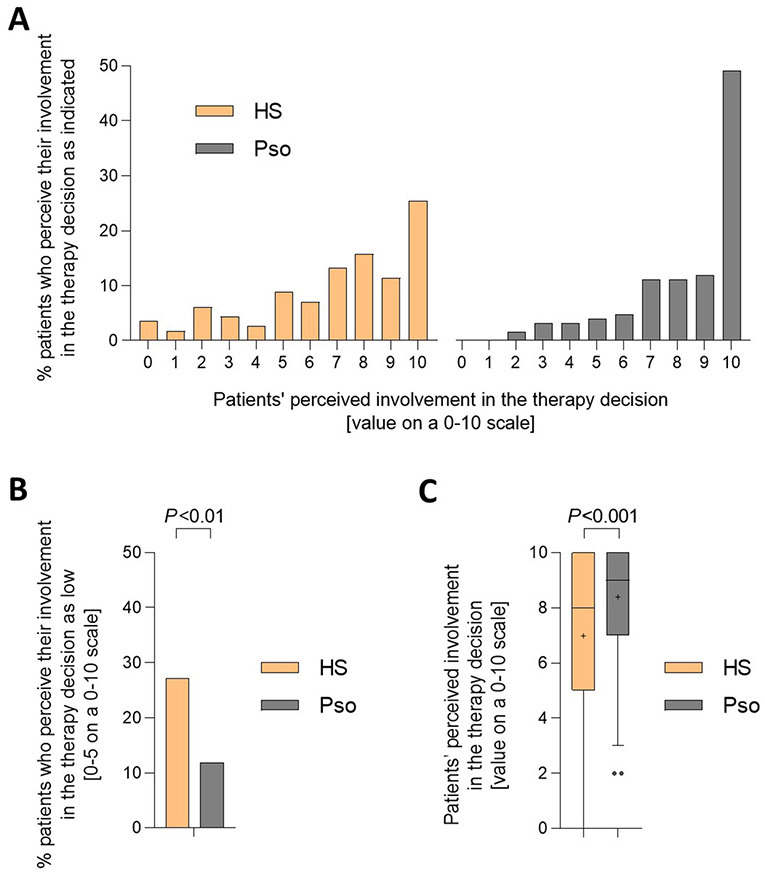
HS patients feel poorly involved in the decision regarding the therapy of their skin disease compared to psoriasis patients. Patients were asked to rate their involvement in the decision regarding the treatment of their skin condition on a scale from 0 (not involved at all) to 10 (completely involved). Responses from 114 HS patients and 126 psoriasis patients were received. **(A)** The bar charts present percentages of HS and psoriasis patients who selected each answer on a scale from 1 to 10. **(B)** The bar chart presents percentages of HS and psoriasis patients who rated their involvement as low, defined as values of 0–5 on the scale. The *P*-value calculated using Pearson χ^2^ test is indicated. **(C)** The levels of HS and psoriasis patients' perceived therapy-decision involvement are presented as Turkey-style box-and-whisker plot, with the maximum length of box whiskers corresponding to the most extreme values in the 1.5-fold interquartile range, outliers displayed as dots, and the “+” representing the mean of the data. The *P*-value calculated using two-tailed Mann–Whitney *U*-test is indicated.

First, we analyzed patients' responses about how involved they felt in the decision-making process of their dermatologist regarding the treatment of their skin condition. Using a scale from 0 to 10, HS patients were more likely than psoriasis patients to select responses indicating low involvement in therapy decision (Figure 1A). In fact, the percentage of HS patients who rated their involvement as low, defined as values of 0–5, was 27.2%, while the percentage of psoriasis patients who gave the same assessment was 11.9% (*P* < 0.01; Figure 1B). Furthermore, about 50% of psoriasis patients rated their involvement in the therapy decision-making process with the highest possible score, compared with only 25% of HS patients (Figure 1A). Consequently, the average degree of perceived therapy decision involvement was significantly lower for HS patients compared to psoriasis patients (mean ± SD: 7.0 ± 2.9 vs. 8.4 ± 2.1; *P* < 0.001; Figure 1C).

### 3.2 Good involvement of HS patients in the decision making regarding their treatment leads to greater patient satisfaction with their therapies

**Figure 2:**
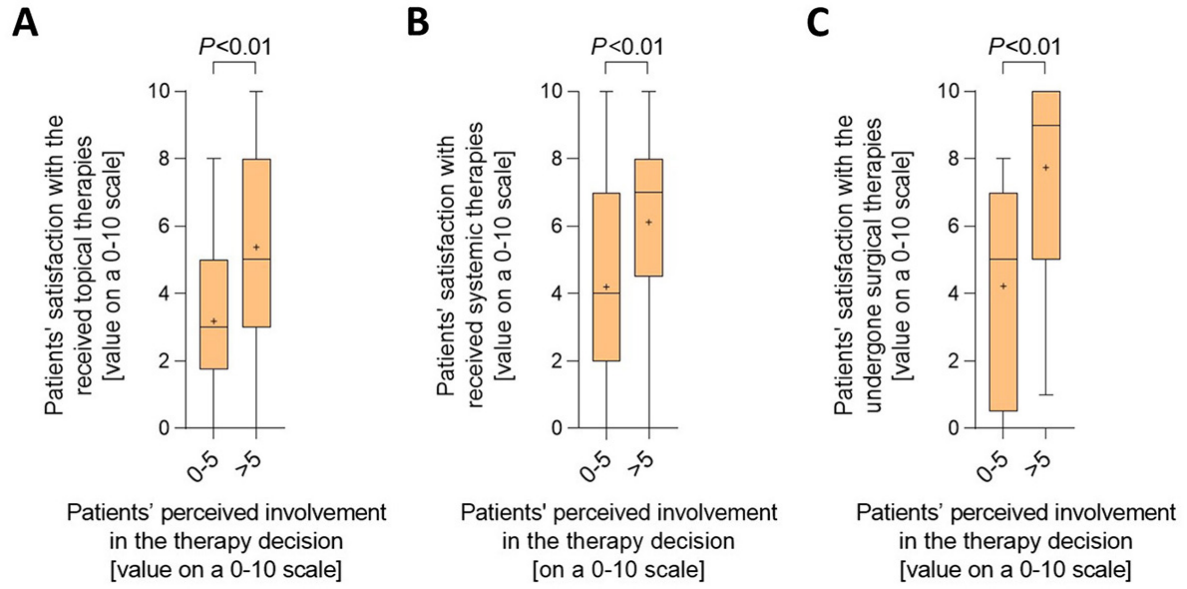
Good involvement of HS patients in the decision making regarding their treatment leads to greater patient satisfaction with their therapies. **(A–C)** HS patients were asked to rate their satisfaction with topical therapies **(A)**, systemic therapies **(B)**, and surgical therapies **(C)** each on a scale from 0 (not satisfied at all) to 10 (completely satisfied). Satisfaction levels were broken down into cases with low (values of 0–5) and moderate to high (values of >5) perceived therapy decision involvement, which they also rated on a scale from 0 (not involved at all) to 10 (completely involved). Answers from 81 **(A)**, 77 **(B)**, and 47 **(C)** patients are presented as Turkey-style box-and-whisker plots, with the maximum length of box whiskers corresponding to the most extreme values in the 1.5-fold interquartile range, outliers displayed as dots, and the “+” representing the mean of the data. *P*-values, calculated using two-tailed Mann–Whitney *U*-test, are indicated.

Next, we evaluated the potential consequences of patients' perceived therapy decision involvement. As demonstrated in Figures 2A–C and Supplementary Figure 1, patients having stated moderate to high therapy involvement were clearly more satisfied with topical and systemic therapies they had received as well as with the surgical therapies they had undergone.

### 3.3 HS patients' perceived involvement in the therapy decision-making process is not associated with demographic, clinical or therapy-related features

**Figure 3:**
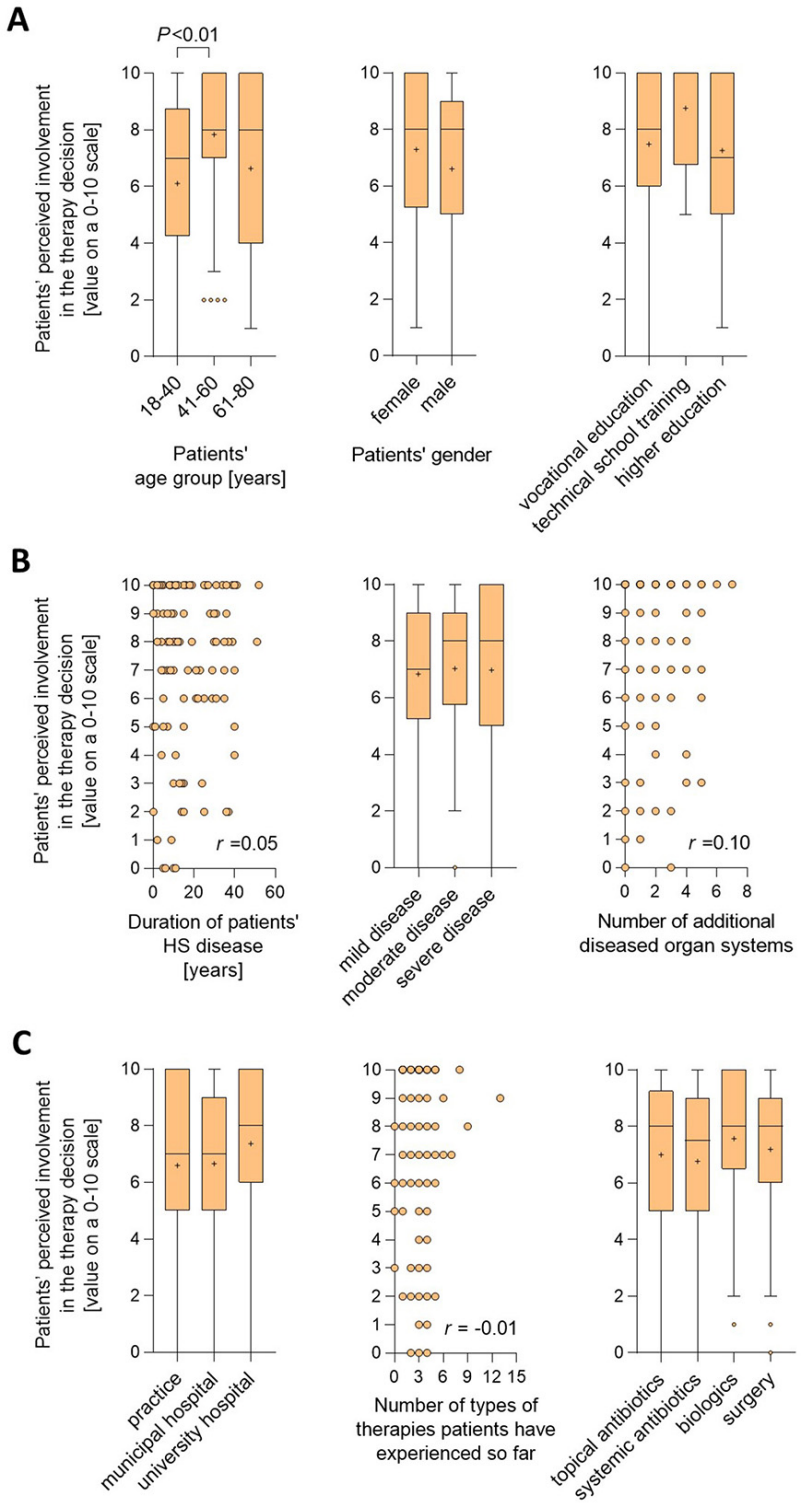
HS patients' perceived involvement in the therapy decision-making process stratified according to demographic, clinical or therapy-related features. Patients were asked to rate their involvement in the decision regarding the treatment of their skin condition on a scale from 0 (not involved at all) to 10 (completely involved). **(A)** Levels of HS patients' perceived therapy decision involvement were broken down into cases with different age groups **(left)**, different gender information **(middle)**, and education background **(right)**. Answers from 114 (age groups), 114 (gender) and 60 (education background) patients are presented as Turkey-style box-and-whisker plots, with the maximum length of box whiskers corresponding to the most extreme values in the 1.5-fold interquartile range, outliers displayed as dots, and the “+” representing the mean of the data. **(B)** Levels of HS patients' perceived therapy decision involvement were correlated with the duration of patients' skin disease **(left)** and the number of additional diseased organ systems **(right)**, and were broken down into cases with different disease severity **(middle)**. Regarding the correlations, answers from 114 patients (skin disease duration and additional diseased organ systems) are presented as X–Y plots, with Spearman's correlation coefficient indicated. Regarding disease severity, answers from 111 patients are presented as Turkey-style box-and-whisker plots. **(C)** Levels of HS patients' perceived therapy decision involvement were broken down into cases recruited at different types of dermatological care facilities **(left)** and cases who had or had not undergone indicated therapies **(right)**, and were correlated with the number of types of therapies patients had experienced so far **(middle)**. Answers from 113 (types of dermatological care facilities) and 111 (undergone therapies) patients are presented as Turkey-style box-and-whisker plots. Regarding the correlation (number of types of therapies received), answers from 111 patients are presented as X–Y plots, with Spearman's correlation coefficient indicated. *P*-values < 0.05, calculated using Mann–Whitney *U*-test (two tailed), are indicated.

We then asked what could have influenced the poor rating of therapy-decision involvement by the patients with HS. To answer this question, we performed subgroup and correlation analyses. Patients in the age range of 18–40 years showed a lower average degree of involvement compared to patients in the age group 41–60 years (*P* < 0.01; Figure 3A). No significant differences were seen regarding the patients older than 60 years, probably because of the low number of cases in this age group. Furthermore, perceived involvement did not vary on gender and educational background.

There was also no association between perceived therapy involvement and the duration of HS, the severity of HS as determined by the dermatologist (considered as mild, moderate and severe disease based on the IHS4 scoring system), or the number of additional organ systems affected (Figure 3B). Likewise, the level of involvement in the therapy decision stated by the patients was not associated with the Hurley stage (score that roughly documents the progression of the disease; comparison of Hurley I vs. II patients: *P* = 0.854; comparison of Hurley I vs. III patients: *P* = 0.371; comparison of Hurley II vs. III patients: *P* = 0.775).

Moreover, neither the type of healthcare facility, where patients were treated, nor the number of types of previously administered therapies had any influence (Figure 3C). Finally, no association was found between perceived therapy involvement and the type of therapies patients had undergone during the past 12 months, although there was a trend toward a perception of better involvement in patients that had received biologics (Figure 3C).

### 3.4 Specific healthcare-related parameters are associated with patients' perceived involvement in the therapy decision-making process

As a further approach to identify factors that could have influenced the poor rating of therapy decision involvement, we performed correlation analyses using the pooled data on specific healthcare-related parameters from both HS and psoriasis patients. Table 2 demonstrates investigated parameters ordered according to the level of significance of their correlation with patients' perceived therapy decision involvement. Significant association was found for the following parameters: the average consultation time with the dermatologist the patients stated (*r*_s_ = 0.16, *P* < 0.05), the waiting time for the first appointment with the dermatologist (negative relationship, *r*_s_ = −0.25, *P* < 0.01), the frequency of visits of the patients to the dermatologist (*r*_s_ = 0.28, *P* < 0.001), and the satisfaction of patients with the information they received from the dermatologist (on a 0–10 scale; *r*_s_ = 0.70, *P* < 0.001). In contrast, no association was found for the number of referrals to other specialties by the dermatologist in the last 12 months and the number of patient visits to the dermatologist to date.

**Table 2:** Healthcare-related parameters that correlate with HS and psoriasis patients' perceived involvement in treatment decisions (pooled analysis).

**Parameter**	***r*_s_**	***P*-value**
Satisfaction with the information from the dermatologist [on a 0–10 scale]	**0.70**	**< 0.001**
Frequency of visits to the dermatologist [number per quarter of the year]	**0.28**	**< 0.001**
Waiting time for first appointment with the dermatologist [weeks]	**−0.25**	**0.006**
Average consultation time with the dermatologist [min]	**0.16**	**0.015**
Number of referrals from your dermatologist to other specialties in the last 12 months	−0.06	0.495
Number of visits to the dermatologist so far	−0.04	0.812

r_s_, Spearman's correlation coefficient. Significant correlations are highlighted in bold.

**Figure 4:**
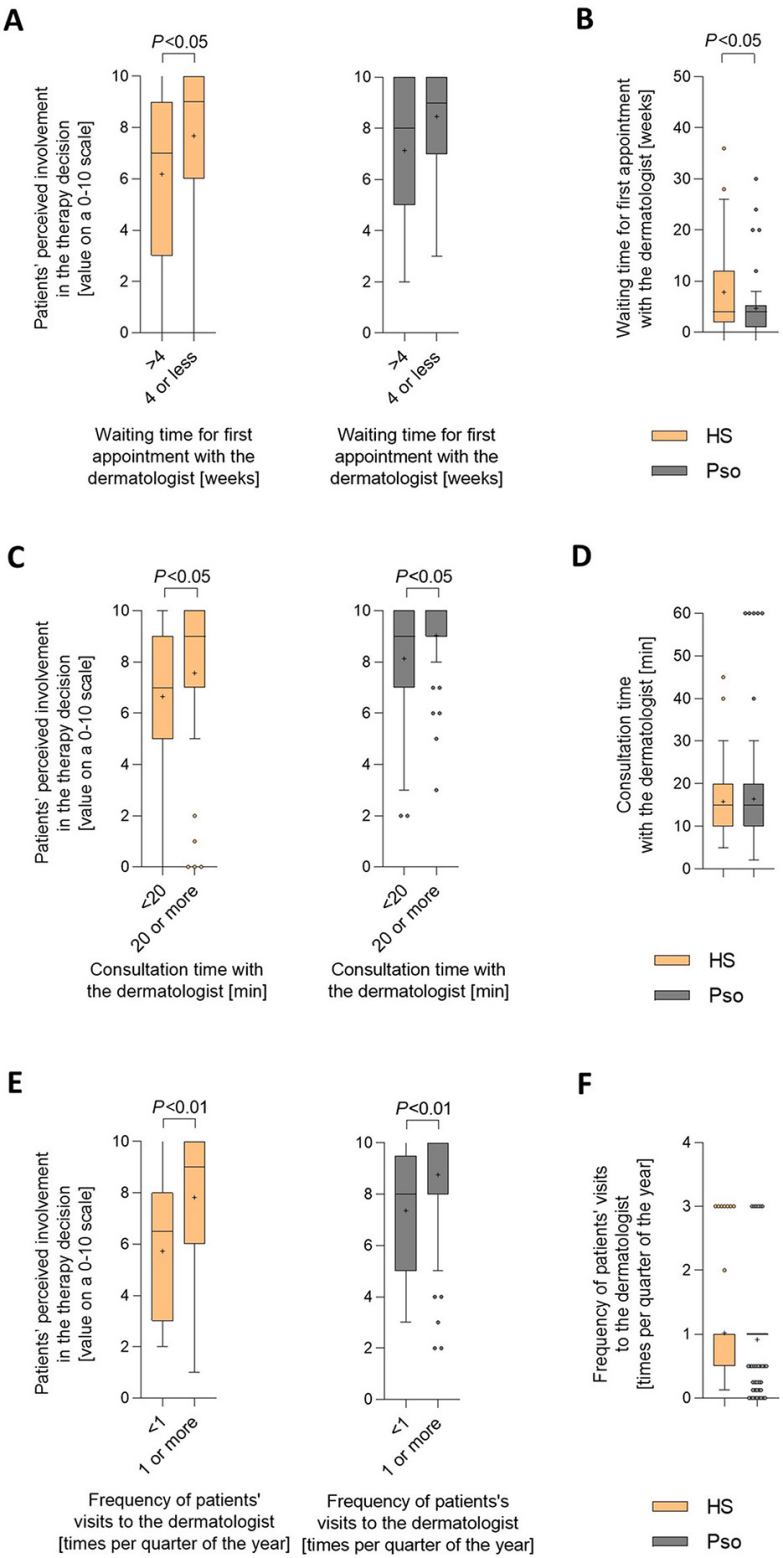
Specific healthcare-related parameters are associated with patients' perceived involvement in the therapy decision-making process. **(A, C, E)** Patients were asked to rate their involvement in the decision regarding the treatment of their skin condition on a scale from 0 (not involved at all) to 10 (completely involved). **(A)** Levels of perceived therapy decision involvement were broken down into cases with waiting times of up to 4 weeks and those with waiting times of longer than 4 weeks. Answers were received from 68 HS patients and 54 psoriasis patients. Data are presented as Turkey-style box-and-whisker plots, with the maximum length of box whiskers corresponding to the most extreme values in the 1.5-fold interquartile range, outliers displayed as dots, and the “+” representing the mean of the data. *P*-values, calculated using two-tailed Mann–Whitney *U*-test, are indicated. **(C)** Levels of perceived therapy decision involvement were broken down into cases with an average length of consultation at their dermatologist visits of at least 20 min vs. < 20 min. Answers were received from 110 HS patients and 122 psoriasis patients. **(E)** Levels of perceived therapy decision involvement were broken down into cases with a frequency of at least once and those with a frequency of less than once in 3 months. Answers were received from 61 HS patients and 108 psoriasis patients. **(B)** Patients were asked to estimate their waiting time for the first appointment with the dermatologist. Answers from 73 HS patients and 54 psoriasis patients are presented as Turkey-style box-and-whisker plots. The *P*-value, calculated using two-tailed Mann–Whitney *U*-test, is indicated. **(D)** Patients were asked to indicate the average length of consultation at their dermatologist visits. Answers from 119 HS patients and 122 psoriasis patients are presented as Turkey-style box-and-whisker plots. The *P*-value was calculated using two-tailed Mann–Whitney *U*-test (no significance). **(F)** Patients were asked to indicate the frequency of their visits to the dermatologist. Answers from 63 HS patients and 121 psoriasis patients are presented as Turkey-style box-and-whisker plots. The *P*-value was calculated using two-tailed Mann–Whitney *U*-test (no significance).

Regarding the waiting time for the first appointment with the dermatologist, HS patients' perceived therapy-decision involvement was higher when waiting times were shorter than 4 weeks compared to those waiting 4 weeks or longer (Figure 4A). Importantly, the waiting time for the first appointment may be partly responsible for the difference in perceived therapy-involvement between patients with HS and patients with psoriasis. In fact, compared to psoriasis patients, HS patients, on average, stated longer waiting time for the first appointment with the dermatologist (mean ± SD: 7.8 ± 8.1 vs. 4.7 ±6.0 weeks; *P* < 0.05; Figure 4B).

HS patients' perceived therapy-decision involvement was higher when average consultation time was 20 min or longer compared to < 20 min (Figure 4C). The same was observed for psoriasis patients. The average consultation time with the dermatologist was comparable for HS and psoriasis patients (mean ± SD: 15.8 ± 8.5 vs. 17.8 ± 19.8 min per visit; Figure 4D). Consequently, the consultation time with the dermatologist is unlikely to be responsible for the difference in perceptions of their involvement in the therapy decision-making process between patients with HS and patients with psoriasis.

HS patients' perceived therapy-decision involvement was higher when they visited their dermatologists one or more times per quarter compared to those visiting them less than once (Figure 4E). The same was seen for psoriasis patients. In line with that, the average frequency of visits to the dermatologist did not differ between HS vs. psoriasis patients (mean ± SD: 1.0 ± 0.8 vs. 0.9 ± 0.6 times per quarter of the year; Figure 4F), and also this parameter is unlikely to be responsible for the difference in perceptions of their therapy-decision involvement between patients with HS and patients with psoriasis.

### 3.5 HS patients' perceived involvement in the therapy decision-making process is strongly associated with the information about HS they receive from the dermatologist

**Figure 5:**
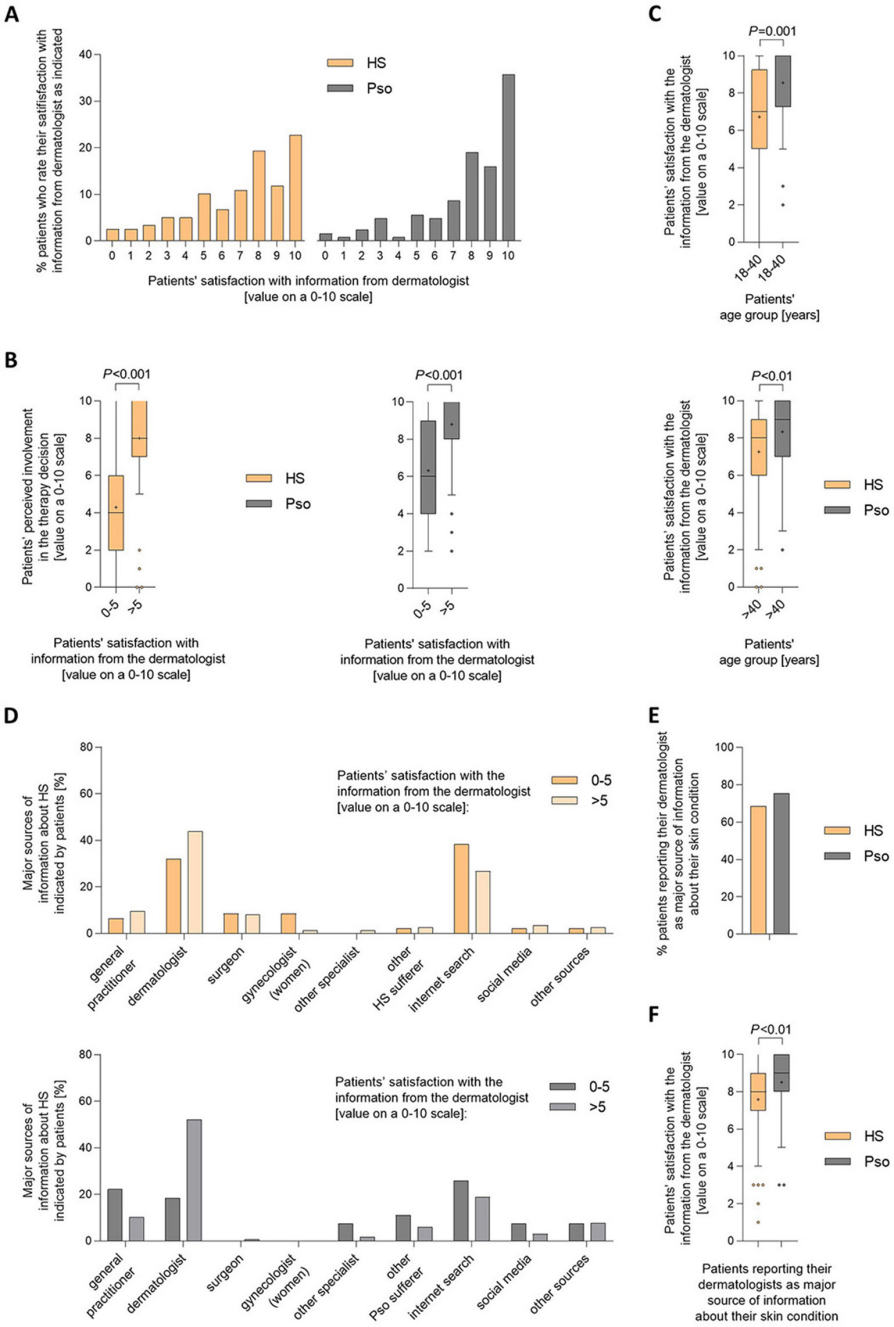
HS patients' perceived involvement in the therapy decision-making process is strongly associated with the information about HS they receive from the dermatologists. Patients were asked to rate their satisfaction with the information from the dermatologist about their skin disease on a scale from 0 (not involved at all) to 10 (completely involved). Answers from 118 HS patients and 126 psoriasis patients were received. **(A)** The bar charts present percentages of HS and psoriasis patients who selected each answer on a scale from 1 to 10. **(B)** Patients were also asked to rate their involvement in the decision regarding the treatment of their skin condition on a 0–10 scale. Levels of HS and psoriasis patients' perceived involvement in therapy decision were broken down into cases that had indicated low (values of 0–5) and moderate-to-high (values of >5) satisfaction with the information from the dermatologist about their skin disease (also rated on a 0-10 scale). Answers from 114 HS patients and 124 psoriasis patients are presented as Turkey-style box-and-whisker plots, with the maximum length of box whiskers corresponding to the most extreme values in the 1.5-fold interquartile range, outliers displayed as dots, and the “+” representing the mean of the data. *P*-values, calculated using two-tailed Mann–Whitney *U*-test, are indicated. **(C)** Answers from HS and psoriasis patients regarding their satisfaction with the information from the dermatologist about their skin disease are presented for patients' age group 18-40 years **(left)** and older than 40 years **(right)** as Turkey-style box-and-whisker plots. *P*-values, calculated using two-tailed Mann–Whitney *U*-test for the comparison of HS vs. psoriasis patients, are indicated. **(D–F)** Patients were asked to indicate the major sources of information about their skin disease they had received (more than 1 answer allowed). Answers were obtained from 115 HS patients and 123 psoriasis patients. **(D)** Answers were broken down into cases with low (values of 0–5) and moderate-to-high (values of >5) satisfaction with the information from the dermatologist about their skin disease, as indicated by the patients based on a 0–10 scale. The percentage of answers is given as bar charts. **(E)** The bar chart presents percentages of HS and psoriasis patients who indicated that the dermatologist was a major source of their information about their skin disease. **(F)** The levels of satisfaction with the information from the dermatologist about their skin disease stated by HS and psoriasis patients on a 0–10 scale was broken down into cases that had or had not indicated that the dermatologist was a major source of their information about their skin disease and are presented as Turkey-style box-and-whisker plots. The *P*-value, calculated using two-tailed Mann–Whitney *U*-test, is indicated.

Since we had found the strongest correlation between patients' perceived involvement in the therapy decision-making process and the information they received about their skin condition from the dermatologists, we looked at this parameter more closely. Using a scale from 0 to 10, patients with HS were more likely than patients with psoriasis to select responses indicating low satisfaction with information provided by their dermatologist (Figure 5A). The rating of therapy-decision involvement by the HS patients significantly increased when they indicated higher (satisfaction values of 6–10) compared to low (satisfaction values ≤ 5) satisfaction with the information received from the dermatologist (Figure 5B and Supplementary Figure 2). The same was observed for patients with psoriasis with regard to the information they received about psoriasis from the dermatologist. It is important to emphasize that, in both younger and older patients, there was a highly significant difference between patients with HS and patients with psoriasis in terms of satisfaction with the information they received from their dermatologist about their skin condition (Figure 5C).

We then wondered whether HS patients, who were dissatisfied with the information provided by their dermatologist, compensated for this by seeking information about their skin disease from other sources. As demonstrated in Figure 5D, a high proportion of HS patients indicated the internet as major source of information. However, no significant difference was found between HS patients with low vs. moderate-to-high satisfaction regarding their dermatologist's information, in terms of viewing the internet as a major source of information about HS. Other medical professionals, such as general practitioners, surgeons, or gynecologists, were cited by up to 10% of patients as their major source of information about HS. Although the total proportion of (female) HS patients stating that they had received relevant information about their skin disease from their gynecologist was relatively low, there was a significant difference between the percentage of those (female) patients with low compared to moderate-to-high satisfaction with the information from the dermatologist (8.5 vs. 1.4%, *P* < 0.05). As a comparison, internet-based information was less frequently stated by psoriasis patients compared to HS patients. Importantly however, about 70%−80% of both patients with HS and patients with psoriasis indicated that their dermatologist was the major source of information about their skin disease (Figure 5E). Within this group, patients with HS were significantly less satisfied with the information they received from their dermatologist about their condition than patients with psoriasis (Figure 5F).

## 4 Discussion

This survey explored the degree of involvement in the decision-making process regarding the therapy of their skin condition as perceived by patients affected by HS and, for comparison, patients affected by psoriasis.

HS is a highly complex disease, considering its different skin lesion types, its progressive, skin-destructive nature, the underlying multifaceted disease mechanisms, and its high prevalence of concomitant diseases (4). The existence of different phenotypes of HS makes the situation even more complex (37). The treatment of HS is very challenging, as the disease goes through different phases with different therapeutic options (4). Drugs for the treatment of HS include recently approved TNF-α and IL-17-targeting antibodies showing at least moderate efficacy, and further developments are underway (4). In addition, a range of substances with little scientific evidence is used (4). Due to often insufficient efficacy and delay in drug treatment, surgical removal of irreversibly damaged skin areas continues to play an important role. The presence of comorbidities, including psychological distress, further increases the complexity of the requirements for therapy decisions for HS patients (4). Thus, in the absence of decision-support algorithms and drug therapies that lead to rapid and complete symptom relief in all patients, HS treatment relies heavily on physician expertise and physician-patient trust. In contrast, psoriasis vulgaris is a skin disease with a clearer pathogenesis (main role of the IL-23-IL-17 pathway), no skin destruction, and effective therapies, making this disease ideal for adequate overall medical care, at least in Western countries (34, 38). In fact, dermatologists are mostly well-trained and several highly effective treatment options and helpful guidelines for their use exist to manage psoriasis patients (39–41). Anti-psoriatic treatments comprise topical agents (for mild cases), phototherapy, and systemic therapies (for moderate to severe cases). The latter include a range of biologics targeting the IL-23—IL-17 pathway and are able to completely reverse symptoms in an important proportion of patients, even with a long disease history (38).

Here, we demonstrated that patients with HS feel significantly less involved in the decision-making process regarding their dermatological therapy than patients with psoriasis. This appears to be highly relevant, as patients with HS who were adequately involved in the decision-making process were more satisfied with the treatment they received, both for topical, systemic, and surgical treatments. We recently demonstrated that the proportion of patients who were satisfied with current medical care for their skin condition was significantly lower in patients with HS than in patients with psoriasis (30.7 vs. 69.4%; *P* < 0.001) (35). There are several reasons why good involvement of HS patients in treatment decisions may improve their satisfaction with the specific dermatological treatment. First, it may improve patients' understanding of their treatment options and outcomes, thereby promoting realistic expectations. Second, patients who feel that their preferences and concerns are considered, may develop greater trust in their doctors. Finally, feeling involved in the management of a chronic and often challenging condition like HS could have a positive impact on patient adherence to treatment.

Regarding possible reasons, the limited involvement in the therapy decision perceived by HS patients was not associated with patients' gender or education. Regarding other demographic factors, the only variable associated with difference was age, with younger patients reporting a lower involvement. However, the evaluation of old patients (>60) was hampered by the small number of cases, which is consistent with the higher prevalence of HS in young and middle-aged people (42, 43). Moreover, neither disease characteristics, nor therapeutic aspects or the type of health care facility had a relevant influence on patients' perceived involvement. Instead, consultation time, frequency of visits to the dermatologist, and waiting time for the first appointment with the dermatologist were found to be linked to therapy decision involvement. The significantly longer waiting time for the first dermatologist's visit for patients with HS compared to patients with psoriasis partly explains the low satisfaction of HS patients with regard to their involvement in treatment decisions. However, the decisive factor in the difference between HS patients and psoriasis patients in terms of their therapy decision involvement appears to be the information that patients receive from their treating dermatologist about their skin condition. Satisfaction with the information received from dermatologists correlated strongly with involvement in treatment decisions in both patients with HS (*r*_s_ = 0.69, *P* < 0.001) and patients with psoriasis (*r*_s_ = 0.65, *P* < 0.001). In this context, it is important to note that patients with HS were generally less satisfied with the information about their skin condition than patients with psoriasis (7.0 ±2.7 vs. 7.9 ±2.45 on a 0–10 scale, *P* < 0.01) (35). Lower satisfaction with the information received from their dermatologists was observed in patients with HS compared to patients with psoriasis, both in younger and older patients, as well as in patients who reported the dermatologist as their major source of information about their skin condition. Lower satisfaction with the information received from their dermatologists about their disease seems to explain why patients with HS feel less involved in treatment decisions than patients with psoriasis. The main reason for this situation is most likely the significantly more complex pathogenesis of HS. In our experience, HS patients are very interested in why they developed the disease, what happens in HS lesions, and what can be done to stop it; however, knowledge about the etiology of HS and the complex immunological processes in the skin lesions often seems limited among dermatologists, including those in university settings. Since many HS patients go through a long and painful period between the first symptoms and diagnosis in Germany, the average delay in diagnosis is around 10 years (44), it is understandable that they are particularly skeptical of doctors. In our opinion, this is also the greatest potential for improving the current situation: the sound immunological training of dermatologists by specialized colleagues. This would not only improve treatment-decision making and therapy adherence of HS patients, but would also benefit patients with other currently unmet skin diseases, as many of these diseases are also chronic inflammatory conditions (45). It should also be noted that with the expected market launch of further new therapeutic approaches for HS, ever greater demands are being placed on the technical and communicative training of dermatologists in the future. Further improvement may be achieved through more frequent and longer visits to the dermatologist, allowing for repeated interactions and extended consultation times. This would give patients more opportunities to receive qualified information about their skin condition and to discuss treatment options in the context of their symptoms, preferences, and concerns. However, this requires structural changes in the healthcare system, including changes to the payment system, which are unlikely to be implemented in the near future. In addition to increasing the quality and quantity of doctor-patient interactions, improving patient information about HS could be done through the creation of information materials that can be accessed via URL links or QR codes, information events for patients, and increased collaboration with HS patient advocacy groups. These do not, of course, replace individual doctor-patient consultations, but can be helpful in a supportive way.

To our knowledge, this is the first study precisely analyzing the real-world level of therapy-decision involvement among patients with HS. To date, there has been only one publication that touched on this topic. It is a paper from 2022 that describes the results of semi-structured interviews that aimed at identifying unmet care needs and important treatment characteristics in the management of HS. Six of the twelve patients involved in the study stated a lack of involvement in the therapy decision-making process (46).

A strength of our study design is that it included different types of dermatological care facilities to represent a broad spectrum of care options and patients, and made a head-to-head comparison with a skin disease with excellent medical care options. In addition, the dermatologist's recording of the severity and treatment of the patient's skin disease is an advantage of the study. Limitations include the fact that not every consecutive patient in the study centers could be recruited for the study and that not every participating patient answered every question, which could potentially have created a bias. Another limitation of the study is that it was only conducted in Germany, so it only takes into account the cultural context of that country. It is possible that HS patients in Germany have a particularly high need for information. For example, for historical reasons, people in Germany may have high expectations of being involved in decision-making processes and do not readily accept opinions. We will therefore, endeavor to encourage colleagues from other countries to conduct similar surveys in their countries.

In conclusion, our study demonstrates limited involvement in the therapy decision-making process perceived by HS patients, which was associated with limited satisfaction with received therapies, and which suggests that improvement can be achieved through training dermatologists on HS and immunological processes by experts in the field.

## Data Availability

The original contributions presented in the study are included in the article/Supplementary material, further inquiries can be directed to the corresponding author.
