# Supplementary material for: Patient involvement in treatment decisions is associated with increased therapy satisfaction in Hidradenitis suppurativa
Source: Front Med (Lausanne). 2025 Sep 3;12:1626345. doi: 10.3389/fmed.2025.1626345 (PMC12440716; doi:10.3389/fmed.2025.1626345)
Supplement: Supplementary file 1 [file Data_Sheet_1.pdf]

Supplementary material

Patient involvement in treatment decisions is associated with increased therapy satisfaction in Hidradenitis suppurativa

Giorgia Cugno,[1,2,3] Sylke Schneider-Burrus,[1,4] Georgios Kokolakis,[1] Dagmar Wilsmann-Theis,[5] Katharina Assaf,[5] Rotraut Moessner,[6] Christian Kromer,[6] Falk G. Bechara,[7] Nessr Abu Rached,[7] Wiebke K. Peitsch,[3] Lisa C. Schneider,[3] Andreas Happ,[8] Valentina Siddi,[9] Diana Kubitzki,[10] Durdana Groß,[11] Markus Friedrich,[2] Staffan Vandersee,[12] Khusru Asadullah,[13] Robert Sabat,[1] Kerstin Wolk[1,*]

[1] Charité – Universitätsmedizin Berlin, corporate member of Freie Universität Berlin and Humboldt-Universität zu Berlin, Translational skin inflammation research and former Psoriasis Research and Treatment Center, Department of Dermatology, Venereology and Allergology, Berlin, Germany

[2] Dermatology practice Dr. Friedrich / Dr. Philipp, Oranienburg, Germany

[3] Department of Dermatology and Phlebology, Vivantes Klinikum im Friedrichshain, Berlin, Germany

[4] Centre for Dermatosurgery, Havelklinik, Berlin, Germany

[5] Centre of skin diseases, University Hospital Bonn, Bonn, Germany

[6] Department of Dermatology, Venereology, and Allergology, University Medical Center, Göttingen, Germany

[7] ICH - International Center for Hidradenitis suppurativa / Acne inversa, Department of Dermatology, Venereology and Allergology, Ruhr-University Bochum, Bochum, Germany

[8] Department of Dermatology, Klinikum Frankfurt (Oder), Frankfurt (Oder), Germany

[9] Dermatology practice Siddi & Bachmann, Berlin, Germany

[10] Medizinisches Versorgungszentrum/Medical Care Center (MVZ) Lobetal, Bernau bei Berlin, Germany

[11] Dermatology practice Dr. Gross, Potsdam, Germany

[12] Department of Dermatology, Bundeswehr Hospital, Berlin, Germany

[13] Dermatology Potsdam Medizinisches Versorgungszentrum/Medical Care Center (MVZ), Potsdam, Germany

Correspondence:
PD Dr. rer. nat. Kerstin Wolk, Translational Skin Inflammation Research, Department of Dermatology, Venereology and Allergology, Charité – Universitätsmedizin Berlin, Campus Charité Mitte | Charitéplatz 1 | 10117 Berlin, Germany; Email: kerstin.wolk@charite.de

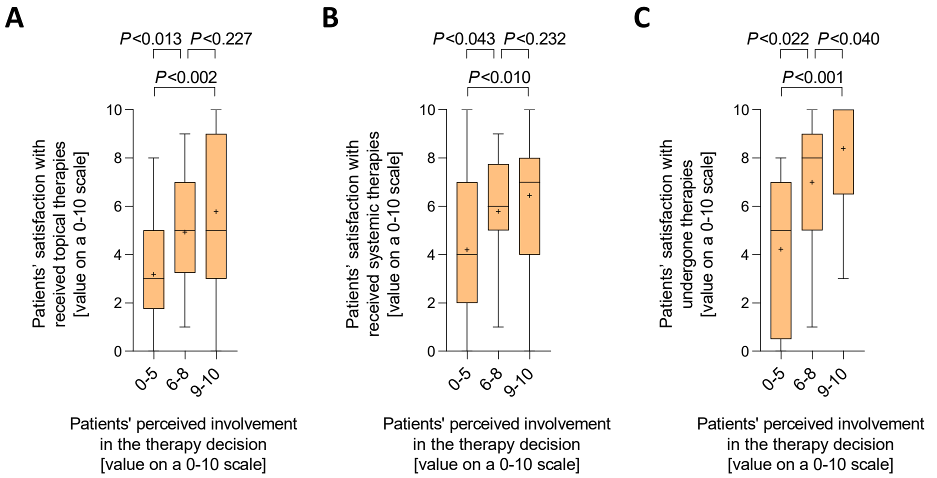

Supplementary Figure 1. Data from Figure 2, with cases broken down into three subgroups. HS patients were asked to rate their satisfaction with topical therapies (A), systemic therapies (B), and surgical therapies (C) each on a scale from 0 (not satisfied at all) to 10 (completely satisfied). Satisfaction levels were broken down into cases with low (values of 0-5), moderate (values of 6-8), and high (values of >8) perceived therapy decision involvement, which they also rated on a scale from 0 (not involved at all) to 10 (completely involved). Answers from 81 (A), 77 (B), and 47 (C) patients are presented as Turkey-style box-and-whisker plots, with the maximum length of box whiskers corresponding to the most extreme values in the 1.5-fold interquartile range, outliers displayed as dots, and the '+' representing the mean of the data. P-values, calculated using two-tailed Mann–Whitney U test, are indicated.

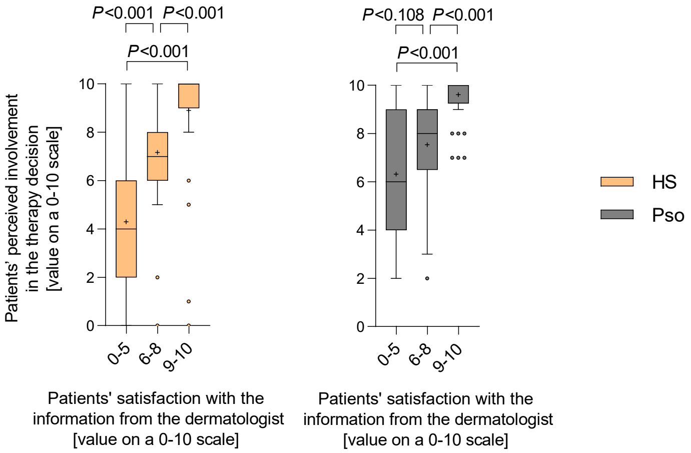

Supplementary Figure 2. Data from Figure 5B, with cases broken down into three subgroups. Patients were asked to rate their involvement in the decision regarding the treatment of their skin condition on a scale from 0 (not involved at all) to 10 (completely involved). Levels of HS and psoriasis patients' perceived involvement in therapy decision were broken down into cases that had indicated low (values of 0-5), moderate (values of 6-8), and high (values of >8) satisfaction with the information from the dermatologist about their skin disease (also rated on a 0-10 scale). Answers from 114 HS patients and 124 psoriasis patients are presented as Turkey-style box-and-whisker plots, with the maximum length of box whiskers corresponding to the most extreme values in the 1.5-fold interquartile range, outliers displayed as dots, and the '+' representing the mean of the data. P-values, calculated using two-tailed Mann–Whitney U test, are indicated.
